# Correlates of quality of life in people living with HIV infection beyond viral load suppression in Africa: a scoping review

**DOI:** 10.1136/bmjph-2025-002824

**Published:** 2026-01-28

**Authors:** Zanele Benedict Nomatshila, Teke Ruffin Apalata, Rachel Crockett, Rachel O’Donnell

**Affiliations:** 1 School of Public Health, Faculty of Medicine and Health Sciences, Walter Sisulu University, Mthatha, Eastern Cape, South Africa; 2 Department of Social Work, Faculty of Law; Humanities and Social Sciences, Walter Sisulu University, Mthatha, Eastern Cape, South Africa; 3 Faculty of Health Sciences and Sport, University of Stirling, Stirling, Scotland, UK; 4 School of Pathology, Faculty of Medicine and Health Sciences, Walter Sisulu University, Mthatha, Eastern Cape, South Africa; 5 Department of Psychology, Faculty of Health Sciences and Sport, Walter Sisulu University, Stirling, Scotland, UK; 6 Institute for Social Marketing and Health, Faculty of Health Sciences and Sport, University of Stirling, Stirling, Scotland, UK

**Keywords:** Epidemiology, Public Health, HIV, Community Health

## Abstract

**Introduction:**

Effective antiretroviral therapy for HIV has allowed people to achieve viral load suppression (VLS), transforming HIV from an acute, life-limiting illness into a chronic condition that is managed effectively with ongoing treatment, reducing it to an undetectable level in the body.

Living with the virus long-term brings new challenges in the management of ongoing treatment and the underlying condition, which may impact quality of life (QoL). However, there is currently no clear evidence regarding factors that promote or predict QoL in this group. This scoping review aimed to identify and synthesise existing literature on the correlates of QoL in patients with HIV and viral load suppressed to identify research gaps and inform future intervention development.

**Method:**

Given the differing healthcare systems internationally and high rates of HIV infection in Africa, the review focused on peer-reviewed research conducted in Africa. ProQuest, PubMed, EBSCO and Sabinet African Journals electronic databases were systematically searched, and 409 studies published between 1 January 2013 and 31 December 2023 were identified for screening. The findings from eight included articles were extracted verbatim for analysis according to four QoL domains: physical, psychological, social and environmental factors.

**Results:**

Poor QoL was associated with increased stigma associated with HIV, lack of family support and poor living conditions, whereas employment and education emerged as correlates of QoL. Demographic factors also influenced QoL, for example, older people with HIV frequently reported low QoL scores, and they were prone to health issues and comorbidities, which had a detrimental influence on their physical and mental health.

**Conclusion:**

Research is required to develop interventions that target medical management and address the social, economic and environmental determinants of QoL among people living with HIV in Africa.

**WHAT IS ALREADY KNOWN ON THIS TOPIC**

- Increasing numbers of people living with HIV (PLWH) are reaching viral load suppression (VLS); however, the challenges of living with the virus after VLS can lead to low quality of life (QoL).
- Research is needed on the correlates of QoL to better understand how to promote a good QoL in those who have achieved VLS.

**WHAT THIS STUDY ADDS**

- This review highlights several factors that are associated with lower and higher perceptions of QoL in PLWH who have achieved VLS in Africa—the first review we are aware of to focus on this geographical location and patient group.

**HOW THIS STUDY MIGHT AFFECT RESEARCH, PRACTICE OR POLICY**

- This review highlights a lack of published research using mixed-methods and qualitative approaches to explore QoL in people with HIV and VLS, indicating a need for future research using a broader range of methodologies to allow a more detailed exploration of the factors that influence QoL.
- This review also highlights limited research focusing on broader dimensions of QoL that go beyond health-related functioning. An increased knowledge of the ways in which social, psychological and environmental factors influence QoL would be beneficial for the development of holistic interventions to improve QoL.

## Introduction

 Globally, there are around 38 million individuals living with HIV/AIDS as of 2020, and more than half of them are from low- and middle-income nations.^1^ Over the past 20 years, there has been significant progress in lowering HIV-related morbidity, death, transmission and stigma and enhancing the health-related quality of life (HRQoL) for those who are living with the virus.^2^ HIV is now considered a chronic, treatable illness due to effective antiretroviral therapy (ART), enabling more people living with HIV (PLWH) to achieve viral load suppression (VLS) through easier access, less complicated dosing and fewer adverse effects.^2^ To assess, monitor and improve HIV testing and treatment, the Joint United Nations (UN) Programme on HIV/AIDS (UNAIDS) set a 90-90-90 target (90% of all PLWH will know their HIV status, 90% of all people with diagnosed HIV infection will receive sustained ART and 90% of all people receiving ART will have viral suppression by 2020) in 2014.^2^ The goal was to improve individual disease outcomes and prevention of disease progression by increasing the percentage of individuals receiving adequate treatment and reaching undetectable HIV levels.^3^ The goal of the three ‘90s has resulted in 27.5 million (26.5 million–27.7 million) individuals on ART by the end of 2020. Frescura and colleagues stated that success has been unequal among nations and demographic groups, and the impact on lowering the number of new HIV infections has been less than early modelling studies had predicted.^4^ A new set of testing and treatment goals for 2025 has been developed in response to the necessity of overcoming obstacles, such as reaching the targets set by the UN, lowering new infections and other barriers mentioned below, to meet the 2030 target of eliminating AIDS as a public health issue, as outlined in the Agenda for Sustainable Development.^5^ Globally, in 2022, 86% of PLWH were aware of their status, 89% of these diagnosed individuals were receiving treatment and 93% of those receiving antiretroviral drugs (ARVs) had VLS.^6^

Research suggests that socioeconomic disparities prevent some PLWH in 11 out of 12 low-income nations in sub-Saharan African countries from getting life-saving HIV testing and treatment or from achieving HIV VLS.^7^ This could be attributed to the fact that individuals with low income frequently face significant barriers to accessing healthcare services, including HIV testing and treatment.^8^ Jaafari *et al*^8^ further attribute this notion to factors such as limited financial resources, lack of transportation, inadequate healthcare infrastructure and lower levels of education, all of which contribute to a reduced awareness of available services and a greater likelihood of experiencing stigma and discrimination within their communities. However, the global report on 90-90-90 targets achievements indicates significant progress in screening, treating and caring for patients living with HIV in regions such as Eastern and Southern Africa, Western and Central Europe, North America, Asia and the Pacific.^9^ According to estimates, Qatar, Botswana, Rwanda, Uganda, Malawi and Slovenia had reached the 90-90-90 targets by 2020; however, Eswatini and Switzerland only achieved just over 86% viral suppression for all PLWH in 2020 when a new and more ambitious target of 95-95-95 for VLS has been set and had been upgraded.^7 9^ According to UNAIDS,^10^ the global number of HIV-infected patients taking ART has increased from 7.7 million in 2010 to 29.8 million by the end of December 2022. In addition, there has been a considerable decrease in the number of new HIV infections and patients who have not participated in ART by 2022. This represents significant progress towards eliminating AIDS by 2030.^9^

Through meeting these goals, a marked increase in the number of PLWH reaching VLS has been observed. While this progress is a remarkable achievement, it means that increasing numbers of PLWH must live with and manage HIV on a long-term basis. However, it has been observed that the challenges of living with the virus after VLS can lead to low overall QoL by introducing an unintended burden of psychological distress and environmental and social challenges.^11^ The phrases quality of life (QoL) and HRQoL are used interchangeably in the literature, although they are different constructs.^12^ HRQoL is defined as the experimentally calculated relationship between health and QoL. QoL includes all aspects of life like psychological being, social interactions, physical health and environmental variables which contribute to the overall QoL.^13^ It is not easy to quantify since it may be expressed differently. Studying QoL is critical in PLWH because living long-term following VLS presents several challenges to QoL.^14^ Patients must live with the side effects of ARV treatment, including increased cholesterol, which increases the risk of non-communicable diseases such as heart disease and diabetes, which are themselves a severe risk to good health.^15^ PLWH face other intense challenges including depression, anxiety, financial stress and HIV-related stigma and discrimination.^16^ On this basis, Lazarus *et al* propose the introduction of the fourth 90% goal, specifically that 90% of patients living with HIV beyond VLS would have good HRQoL.^11^

However, only a few studies^17 18^ assessing the QoL of PLWH have been conducted in Africa. These studies have limitations, including the use of non-representative samples and consideration of measures across multiple interacting life domains, which make it impossible to isolate the effects of VLS on QoL. There is a need for good evidence on the correlates of QoL to support future research on promoting a high QoL in those who achieve VLS. This would help ensure that patients who have HIV VLS can enjoy life fully; those diagnosed with HIV can enjoy good as well as long lives. The objective of this scoping review was to identify and synthesise existing literature on the correlates of QoL in people with HIV and VLS and identify any literature gaps.

## Methods

A scoping review methodology was employed due to the lack of a clear overview of the evidence concerning QoL following VLS. This type of review concentrates on identifying research gaps.^19^ A six-stage scoping review framework was used.^20^

### Stage 1: identifying the research question

Arksey and O’Malley^20^ recommend using a broad approach to determine the research question to achieve thorough coverage. Our research question was framed as follows: *What is the current research landscape regarding the QoL of HIV-positive patients in Africa who have achieved VLS, and what factors influence their QoL outcomes?*^20^ They also emphasise the need to define terminology at the start of scoping reviews. For consistency, we used the broad definition of QoL per Veenhoven^21^ as a subjective evaluation impacted by several elements, including physical health, psychological state, social interactions and environmental circumstances.^21^ It acknowledges the complexities of QoL as a multidimensional construct that includes both personal and societal aspects. References to QoL in the context of health and disease as HRQoL followed the CDC.^9^ Focusing on individuals who have achieved VLS gives unique insights into the factors that influence QoL in this population, which may be used to guide efforts to improve the health and well-being of all patients with HIV.

### Stage 2: identifying relevant studies

**Figure 1:**
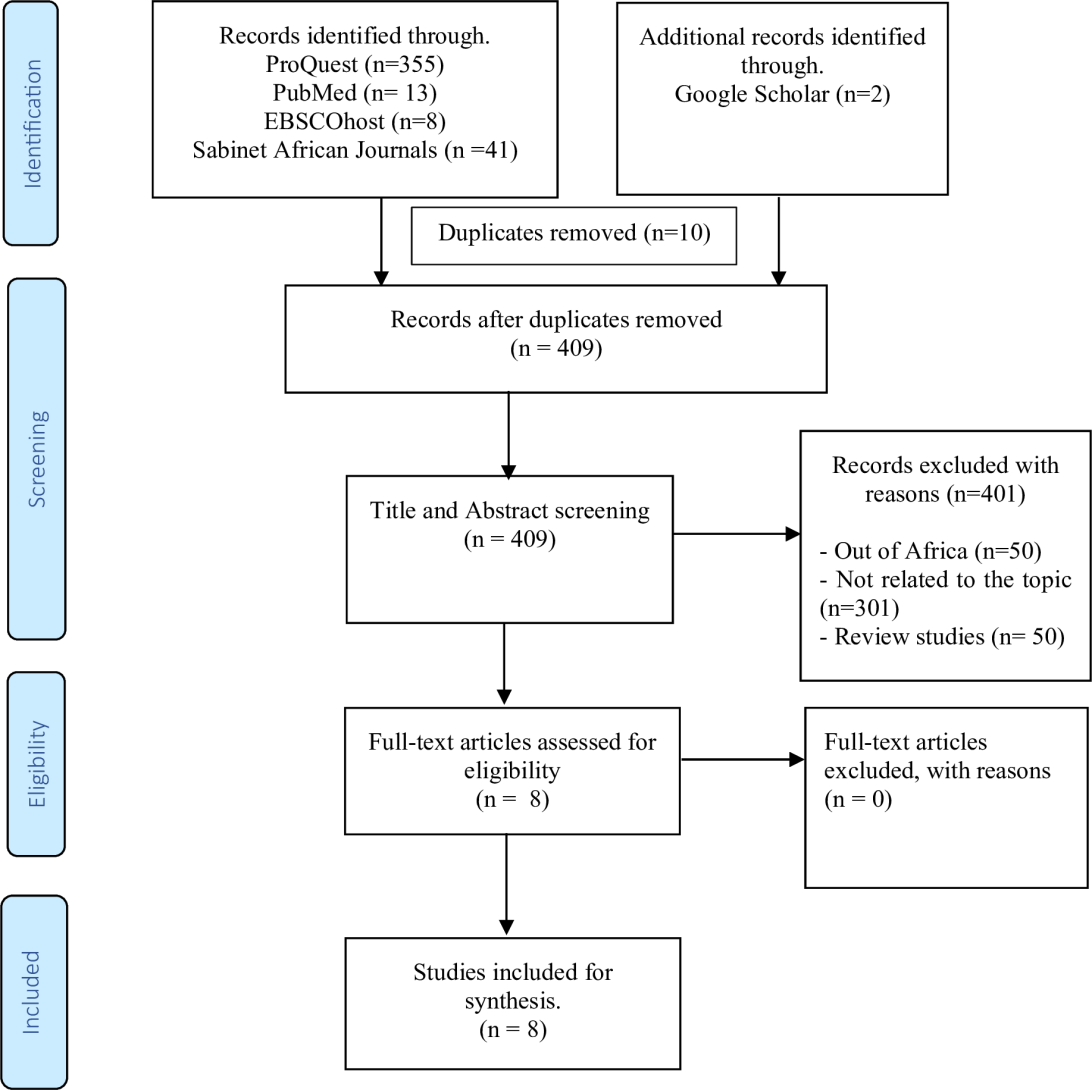
Flow chart of study selection depicting sources of evidence included in the scoping review.^28^

Guided by the Preferred Reporting Items for Systematic Reviews and Meta-Analyses extension for Scoping Reviews (PRISMA-ScR) (see figure 1), four electronic databases—ProQuest, MEDLINE on EBSCO, the PUBMED Health Source-Consumer Edition and the local Sabinet African Journals database—were searched, between January 2013 and December 2023, using the following search strategy: (HIV OR “human immunodeficiency virus” OR AIDS OR “acquired human immunodeficiency syndrome”) AND (HRQoL OR “quality of life”) AND (“viral load suppression” AND Africa). To further enhance the comprehensiveness of the search, Google Scholar was employed to conduct both backward and forward citation searches on the initial citations identified for inclusion in the review (see table 1). The results from these searches were exported to EndNote (V.20), and duplicate entries were subsequently removed.

**Table 1:** Information sources for scoping review on correlates of QoL

Database	Search terms	Limitations
ProQuest Central	(“health-related quality of life” OR HRQoL) AND “HIV” AND “viral load suppression” AND Africa	Limited by: Peer reviewed Narrowed by: Entered date: 1 January 2013 to 31 December 2023
PubMed	Search: HIV) OR (“human immunodeficiency virus”)) OR (AIDS)) OR (“acquired human immunodeficiency syndrome”) AND (HRQoL) OR (“quality of life”)) AND (“viral load suppression”) AND (AFRICA)	Filters: from 1 January 2013 to 31 December 2023
EBSCOhost (MEDLINE and Health Source: Consumer Edition)	(HIV OR “human immunodeficiency virus” OR AIDS OR “acquired human immunodeficiency syndrome”) AND (HRQoL OR “quality of life”) AND “viral load suppression” AND Africa	Limiters—peer reviewed; publication date: 1 January 2013 to 31 December 2023 Expanders—apply equivalent subjects Search modes—Boolean/phrase
Sabinet African Journals	[All: HIV & AIDS] AND [[All: HRQoL] OR [All: quality of life]] AND [All: viral load suppression]] AND [Africa]]	Limiters: 1 January 2013 to 31 December 2023

AIDS, Acquired Immunodeficiency Syndrome ; HIV, Human Immunodeficiency Virus ; HRQoL, health-related quality of life; HRQoL, Health Related Quality of Life; MEDLINE, Medical Literature Analysis and Retrieval System Online; QoL, quality of life; SABINET, South African Bibliographic Network.

#### Information sources for scoping review on correlates of QoL

The search strategy for this review on the correlates of QoL is summarised in table 1 above.

### Stage 3: data extraction

Three authors (ZBN, RO and RC) independently extracted data from various studies using a standardised charting form created in Microsoft Excel. The research team developed this form specifically to meet the aims of the review. The collected data were systematically arranged and comprised specific details, including extractor/reviewer, date of extraction, title and abstract; bibliographic details such as author, URL, inclusion status and reasons for exclusion; characteristics of the study, including the country, setting, aim and design; participant characteristics, providing a description of the sample and population; QoL information detailing the domains and instruments used; and correlates of QoL. This approach ensured accurate and consistent capture of all relevant information.

### Stage 4: selection of sources of evidence 

Records were first screened at the title/abstract stage against inclusion and exclusion criteria developed by the authors (see table 1). The authors conducted screening of the extracted data to reach an agreement. There were no areas of disagreement in the process, and RC and RO independently checked 10%. Articles were included if they measured QoL on ART beyond VLS and were peer-reviewed publications that presented original or primary data written in English. Animal studies, non-full-text papers that presented secondary data, were excluded from this review. The remaining articles were single-screened using full text by the first author.

#### Eligibility criteria

The inclusion and exclusion criteria are summarised in table 2 below.

**Table 2:** Inclusion and exclusion criteria

Criterion	Inclusion	Exclusion
Population	Patients with HIV living in Africa who have achieved VLS or where VLS status was analysed as a correlate of QoL	Patients living with HIV in Africa who have not achieved VLS, and with illnesses other than HIV outside Africa
Design	Qualitative, quantitative and mixed-method designs	Review studies, protocols and grey literature
Context	Studies conducted within the African continent	Studies conducted outside the African continent
Language	Studies published in the English language	Studies published using languages other than English
Period	Studies conducted from January 2013 to December 2023 (10-year projection)	Studies conducted before January December 2013 and after 2023

QoL, quality of life; VLS, viral load suppression.

### Stage 5: data charting

All records were saved to Endnote for screening. Data relevant to the research question were extracted from the articles included and tabulated in Microsoft Word to identify and describe the correlates of each of the four QoL domains. Tabulation also allowed the relationships between correlates, and outcomes to be mapped.

### Stage 6: collating, summarising and reporting results

An article, specifically a peer-reviewed journal article, serves as our unit of analysis. To present the range of research identified, we grouped the articles according to the different headings during data charting. We then evaluated the outcomes for their relevance to the aims of our scoping review.

## Results

### Methods of handling results

The PRISMA-ScR was used for reporting purposes (see checklist in online supplemental file 1).^22^ A total of 409 records from academic literature databases were obtained after removing duplicates (see figure 1). Following the screening process, we included eight peer-reviewed journal articles for analysis. While the main focus was on populations with VLS, however, studies were also included if they reported on QoL in groups where a significant proportion of participants had VLS, or if VLS status was examined as a correlate of QoL. This approach was taken due to the limited number of studies specifically involving populations with VLS in Africa.

Online supplemental table S1 provides an overview of the eight studies included in the review. Most of the articles that met the inclusion criteria used quantitative methods;^1718 23^,^28^ some used cross-sectional surveys,^1823^,^25 27 28^ with one randomised controlled trial^26^ and one cohort study.^17^ No mixed-methods or qualitative studies were identified in this review, which signifies a research gap. Geographical locations included Botswana,^23 24^ Tanzania,^18 25^ Ethiopia,^27^ South Africa^26 28^ and Uganda.^17 26^ A greater number of studies were conducted with women^1718 23^,^28^ than with men.^24 26 27^ Of the identified articles, participants between 55 years and above were less often studied.^25^ Age and sex were the most studied correlates of QoL and HRQoL^17 18 24 26 28^ followed by education status,^24 26 27^ and access to service.^25 27^

Online supplemental table S2 summarises the correlates of QoL studies in each article. Interestingly, six of the included articles focused only on HRQoL^23^,^28^ compared to only two studies that focused on overall QoL.^17 18^ Most of the included articles measured the physical QoL.^1718 23^,^28^ Only a few articles (three articles) did not explore social QoL^1725^,^28^ and/or environmental QoL.^18 26 27^ There was an equal split between included articles on associations between education and HRQoL, wherein 50% found association^17 18 23 26^ and 50% found no association.^24 25 27 28^ Being in employment was associated with a good QoL.^23 24 27^

The robust selection of sources of evidence included in the scoping review is demonstrated in figure 1 (image).

### Synthesis of results

#### Correlates of QoL

##### Psychological factors that impact QoL

One article reported that positive mental health (as measured by the mental health scale (MHS)) improved QoL among PLWH on ART, with females experiencing lower mental health compared with males.^17^ Moreover, it was reported that depression was negatively associated with physical health regardless of ART status.^17^ Five studies suggest that QoL is negatively impacted by stigma and discrimination.^17 24 24 27 28^ Additionally, one study found that using the Internet, watching television and owning a bank account are associated with better physical and mental health QoL scores.^26^

##### Social factors that impact QoL

Lack of family support and insecure living conditions among PLWH were found to be associated with poor HRQoL.^24^ Lack of sexual activity was found to affect social QoL.^17 18 23^ The use of drugs,^17 28^ access to services such as time spent at clinics^25^ and homelessness^28^ were found to decrease QoL.

##### Environmental factors that impact QoL

Women living in areas where males are dominant in terms of power and cultural norms tend to report lower HRQoL, suggesting that the environment in which they live can significantly impact how they perceive their health and overall well-being.^25^ Urban-based clients reported a higher HRQoL than rural counterparts, potentially due to differences in living conditions and access to resources between urban and rural areas.^25 27^ The analysed studies revealed that HRQoL declines with age, with younger respondents exhibiting higher HRQoL scores than older respondents across all domains.^25 28^ Females have shown relatively higher psychological health than males after receiving highly active antiretroviral therapy (HAART), suggesting gender differences in QoL outcomes.^17 27^

##### Socioeconomic factors that impact QoL

Two studies suggested that the impact of employment on HRQoL is generally favourable.^23 24^ However, a third study found no relationship between HRQoL and employment and income.^25^ Other studies suggest that better financial status, higher income per month, infrastructure and support systems increase QoL, particularly in people living in urban areas.^17 27^ Several studies suggest that higher educational status positively relates to HRQoL and that individuals with a higher level of education exhibit higher overall QoL and physical health.^17 23 24 26 27^ One study suggested that a lack of formal or primary education is associated with poor QoL.^17^

##### Physical factors that impact QoL

Several studies suggest that the physical factors, such as relatively low pain, feeling reasonably energetic, sleep satisfaction and VLS, increase QoL.^1723 24 26^,^28^ Interestingly, one study suggests that comorbidities are not associated with poor QoL.^18^ However, one study posits that VL is strongly associated with QoL.^23^ The presence of HIV-related symptoms, particularly among those in WHO stages 3 and 4, was associated with poor QoL.^17^

## Discussion

Most of the eight articles included in this review focused on the measurement of HRQoL, as measured by physical indicators, with several examining how health conditions affect HIV-positive individuals’ daily lives. Only two included studies focused on the measurement of QoL, focusing not only on health-related variables but also on social, economic, environmental and psychological factors contributing to QoL. The terms HRQoL and QoL were often used interchangeably in these articles, despite the conceptual differences between them. HRQoL refers to characteristics of QoL that are directly influenced by and connected to one’s physical and mental health.^9^ Several authors have emphasised the significance of HRQoL in achieving long-term health and well-being in PLWH, urging health systems, international societies and those providing AIDS services to include it as an essential aim of HIV care.^29^ As HIV is increasingly considered a chronic condition rather than a fatal illness, it is important that QoL studies in this patient group include measurement of physical, mental and social well-being, as these are also critical in the holistic care of patients with HIV.

All eight included articles in this review used quantitative methods,^1718 23^,^28^ highlighting a lack of published research using mixed-methods or qualitative approaches. Qualitative studies play a crucial role in comprehending the factors and relationships that affect QoL among patients with HIV. They provide valuable perspectives on individual experiences, contextual factors, barriers to care and support for well-being.^30^ This understanding is vital for developing targeted treatments and policies aimed at enhancing the overall QoL for those living with HIV.

This review revealed that age and sex are the most common correlates of QoL.^1823^,^25 27 28^ As people age, they are more prone to encounter age-related health difficulties and comorbidities, such as cardiovascular disease, diabetes and other chronic illnesses. The study conducted in New York between 2011 and 2014 revealed that older persons living with HIV frequently have a more significant burden of these diseases than their HIV-negative colleagues, which can considerably influence their overall QoL.^31^ Age has been shown to impact treatment adherence patterns. Older persons may struggle with the complications of managing long-term ART regimens, particularly if they are also coping with drugs for other health concerns. Age is a significant predictor of QoL in HIV-positive patients because it interacts with health status, psychological characteristics, treatment adherence, social support networks and worries about future health outcomes.^32^ One study on challenges and coping strategies among young adults living with perinatally acquired HIV infection in Botswana indicated that the majority of young adults living with perinatally acquired HIV (YALPH) had suppressed their HIV viral load and considered themselves to be in good physical condition and functioning.^23^ However, they face numerous challenges, including intermittent or longstanding poor ART adherence, disabilities and impairments, poor school performance and attainment, unemployment, financial stressors, fear of stigma, disclosure worries and concerns, and limited social support, which put them at high risk of QoL.^23^ These findings call for deliberate action to identify and help YALPH’s most vulnerable subgroups attain access to education, employment, livelihood, psychological and other necessary supports. Furthermore, addressing these age-related problems is critical to enhancing the QoL of PLWH as they age.

Sexual health and experiences have a substantial impact on the QoL of HIV-positive patients.^17 18 24 27 28^ According to studies, sexual issues and dysfunctions are more frequent among HIV-positive persons than in the healthy population, regardless of gender.^33^ The study conducted by Milewska-Buzun *et al* in Poland established that HIV-positive individuals who express higher happiness with their sexual encounters tend to report better overall QoL.^34^ On the contrary, the study conducted in São Paulo on sexual dysfunctions among PLWH with long-term treatment with ART found that PLHIV on long-term ART therapy showed worrisome rates of depression/anxiety, which is linked to sexual and physical health issues.^35^ The fear of transferring the virus, anxieties about sexual performance and stigma associated with being HIV-positive can further worsen these challenges, resulting in a negative feedback loop harming mental health and overall life satisfaction. The capacity to participate in intimate relationships is critical for emotional support and social connectivity. Positive social connections and supportive relationships are vital for boosting QoL; hence, challenges in managing sexual relationships can contribute to feelings of isolation and decreased life satisfaction.^34 36^ This study reports that sex is an important factor in the QoL of HIV-positive patients, in addition to viral load reduction, psychological well-being, social interactions, stigma and coping methods, in line with the literature.^17 18 23 37^ Addressing these issues is critical for improving the overall health and life satisfaction of those living with HIV. Comprehensive treatment, including attention to sexual health, is essential for promoting resilience and improving the QoL for this group.^38^

Few studies assessing the QoL of PLWH have taken into account the multidimensional contribution and interaction of various life domains that may influence QoL concurrently, and only a few include a representative sample.^39 40^ This study revealed that individuals living with HIV have poor psychological QoL as compared with physical health, level of independence and spirituality.^27^ This review sought to identify and synthesise existing literature on the correlates of QoL in patients with HIV and VLS and identify any literature gaps. The study conducted in Nigeria and South Africa recorded similar scores across the spiritual, religious, personal beliefs, physical and psychological health domains.^41 42^ In corroboration, Mutabazi-Mwesigire and colleagues underscored that the domain of mental health, particularly in females, improves when they are on ART treatment, which indicates a need to combine psychological interventions with ART to achieve positive mental health.^17^ In addition, individuals who had depression reported lower physical health scores as compared with those who did not have the mental disorder, regardless of their ART status.^17^ This supports the findings of Langebeek and colleagues, indicating that PLWH report poor physical and mental health when they are depressed.^43^

QoL is negatively impacted by stigma and discrimination, as well as poor psychological, social and emotional well-being, as supported by literature.^18 24 28^ This is further reported by several researchers who identified stigma as a potential threat to the QoL of PLWH.^44 45^ Apart from stigma, the lack of family support and insecure living conditions among PLWH also prove to have a negative influence on HRQoL in line with the findings by other authors.^24^ Social support is crucial to the QoL of PLWH because the more they converse with others, the more they feel positive towards life.^46 47^

### Socioeconomic factors

In the sampled articles, employment appeared to improve HRQoL,^24^ whereas Okere and colleagues found that the level of employment and income is not associated with HRQoL.^25^ In a study conducted by Miners and colleagues, it was argued that using employment variables as a determining factor for QoL may be problematic because poor health may cause unemployment as much as employment can positively affect QoL.^48 49^ For instance, some patients with HIV who are already working may suffer from discrimination from their coworkers, leading them to lose their jobs; meanwhile, others may find their working environments to be safe and nurturing, which will eventually increase their HRQoL.

Education is a gateway to better-paid employment, and therefore, one approach to supporting younger people with HIV to have good long-term QoL might be to provide additional support to help them complete their education. The present review found that self-education^24^ and higher educational status positively influence HRQoL, and individuals with higher education levels scored high on global QoL and Physical Health Summary.^16 18 23 26 27 50^ This is in line with a study conducted by Degroote and colleagues, which revealed that a higher educational level in PLWH is associated with higher QoL.^50^ On the other hand, the lack of formal or primary education was negatively associated with QoL^18^ and education of less than 5 years is linked to poor mental health and QoL.^50^ Furthermore, factors such as improved financial status, higher monthly income, infrastructure and support systems, especially for people living in urban areas, enhanced the QoL.

### Nexus of demographic and environmental factors

The analysed studies revealed that HRQoL declines with age, with younger respondents exhibiting higher HRQoL scores compared with older respondents across all domains.^25 28^ This is in line with a study conducted by Robberstad and Olsen,^51^ which underscored that the more people age, the more their HRQoL declines. Old age is generally associated with poor physical health; however, the authors have shown that mental health is less dependent on age.^50^ In this review, it was reported that females have relatively higher psychological health than males after receiving HAART, suggesting gender differences in QoL outcomes.^17 27^ Moreover, the review found that women staying in male-dominated areas are likely to report low HRQoL, suggesting the influence of environmental factors on QoL outcomes.^25^ It was also found that people residing in urban areas have higher HRQoL than their rural counterparts, indicating differences in living conditions and access to resources between urban and rural areas.^25^

### Strengths and limitations of the study

Including only English-language publications may have influenced the findings, and by limiting our searches to include only published studies, we may have overlooked other relevant research available as ‘grey’ literature. Furthermore, we screened potentially relevant publications only by title and abstract in the early stages of the review process. To ensure that all studies meeting the inclusion criteria were not excluded, we employed a liberal approach to include all records that we initially doubted met the inclusion criteria. We also broadened our search to include broader keywords during the initial screening. Individual papers were not evaluated in terms of quality because this was a fast-scoping review with an inclusive approach. Reports were not critically assessed during this scoping review because critical appraisal and risk of bias evaluations are not required components of scoping reviews. The primary goal of a scoping review is to identify and synthesise existing literature rather than to assess the quality of individual studies in depth.^52^ Our review included papers published up until 31 December 2023. Due to time and resource limitations, we were not able to update our database search prior to publication. In addition, our scoping review methods do not align with those presented in the Joanna Briggs Institute Manual for Evidence Synthesis associated with scoping reviews, as our protocol and database searches were completed prior to its publication. Future review work in this topic area could be undertaken to address these limitations.

This scoping study on the correlates of QoL for HIV-positive patients who have achieved VLS is helpful since it takes a holistic approach to understanding the varied factors that influence well-being. The findings of this scoping review can be used to inform future research, building on the research gaps identified, which in turn could inform more holistic development of interventions designed to improve QoL in this patient group.

## Conclusion

This scoping review highlights very few published studies which (a) focus on assessment of broader dimensions of QoL that go beyond physical functioning or (b) use mixed-methods or qualitative approaches to better understand the complexities of QoL in persons living with HIV and VLS in Africa. Individual studies suggest that HIV-related stigma, lack of family support and poor living conditions may impact negatively on QoL in people with HIV and VLS, whereas employment and education may impact positively. Research is required to better understand the factors which influence QoL perceptions, including age, gender and location (rural vs urban living). This would assist in developing holistic, targeted interventions that go beyond medical management and seek to improve QoL in PLWH and VLS.

## Supplementary material

10.1136/bmjph-2025-002824 online supplemental file 1

## Data Availability

No data are available.
