# Supplementary material for: Correlates of quality of life in people living with HIV infection beyond viral load suppression in Africa: a scoping review
Source: BMJ Public Health. 2026 Jan 28;4(1):e002824. doi: 10.1136/bmjph-2025-002824 (PMC12853436; doi:10.1136/bmjph-2025-002824)
Supplement: online supplemental file 1 [file bmjph-4-1-s001.docx]

**Table S1: Characteristics of sources included in the scoping review**

| **Article** | **Geographical Location** | **Participants** | **Research design** | **Study Aim** | **Assessment methods and tools** | **Assessed correlates of QOL** | **Assessed Domains of QoL** |
| --- | --- | --- | --- | --- | --- | --- | --- |
| Karugaba, et al. ^23^ | Botswana-Baylor Children’s Clinical Centre of Excellence. | Five hundred nine female adults aged 18-30 years. 255 living with perinatally acquired HIV. | Cross-sectional survey conducted from April to July 2019 | To assess HRQOL and its determinants among young adults living with HIV (YALPH). | Interviews and questionnaires, including WHOQOL-HIV BREF | Sociodemographic, HIV care, and service access | Physical, emotional, functional and global well-being; and cognitive functioning using the Function Assessment of HIV infection (FAHI) |
| Ndubuka^24^ | South-eastern Botswana Public Clinics | Four hundred fifty-six participants, aged 15-56 years, females=310, males=146. |  | To assess the HRQoL of patients who had had ART for at least five years | Interviews using a 59-item structured interview schedule and the WHOQoL–HIV–BREF instrument. | Age, employment, health status, coping experience, coping with life, self-education on HIV, sex with more than one partner in the previous 12 months. | Physical, psychological, independence, social relationships, environmental, spirituality, and understanding circumcision. |
| Okere, et al. ^25^ | North-western Tanzania  Clinics and differentiated delivery clinics/clubs (DSD) | 629 participants aged 25 + years, males=233 and females=396, | Cross-sectional survey from May-August 2019 | To assess HRQoL among ART clients | Kiswahili  translation of the Function Assessment of HIV Infection (FAHI) tool, in addition to socio-demographic, HIV care, and service accessibility data. | Sociodemographic and services access | Physical well-being has ten items, emotional well-being has ten items, and functional and global well-being has ten items. |
| Perez, et al. ^26^ | Infectious Diseases Institute and the University of Cape Town in Uganda and South Africa | 203 HIV+ pregnant women aged 18 years and above | Randomised, open-label trial | To Assess HQRoL as part of the DoLPHIN -2 study that randomised pregnant women initiating ART in the third trimester. | HIV Medical Outcomes Survey (MOS-HIV) | Age, marital status, educational level, viral load, CD4 count treatment duration | Physical and mental health outcomes, general QoL |
| Surur, et al. ^27^ | North-western Ethiopia Health facility-based | 400 HIV/AIDS patients on highly active anti-retroviral  Therapy, 181 males and 219 females aged above 30 years | Quantitative Cross-Sectional Study | To assess HRQoL of HIV/AIDS patients and the association of socio-demographic and disease-related variables with health-related quality of life | The pre-tested semi-structured questionnaire was adapted from the WHOQoL-BREF instrument. | Sex, Age, current illness, CD-4 count, educational status, marital status, and WHO clinical stages | -Physical health  -Psychological health Level of -independence -Social relationship  -Environment health  -Spirituality Health  -Overall perception of HRQoL |
| Wang, et al. ^28^ | Durban, South Africa | 1,363 Female Sex Workers (FSW) aged 18+ diagnosed with HIV | Adaptive  randomised intervention trial, quantitative approach | To examine HRQoL across  the five EQ-5D domains and identify characteristics associated with HRQoL scores among FSW living with HIV | HRQoL information was collected using the EQ-5D-3L tool | Age, Current ART status, viral load, Drug use, Homelessness, Experience of physical or sexual violence, stigma, | Summary HRQoL score, representing health utility |
| Mutabazi-Mwesigire, et al. ^17^ | Uganda urban clinics | 1 274 enrolled, 640 participants receiving ART, and 634 not eligible but received primary care,  220 males and 420 females aged between 30 and 40+ | Prospective Cohort study, quantitative research | To investigate factors associated with QoL among PLHIV receiving primary care and those on ART. | A pre-tested semi-structured questionnaire, which was adopted from World Health Organization Quality of life brief instrument, | Sex, age, income, religion, depression and opportunistic infection | Physical domain |
| Massawe, et al. ^18^ | Dar es Salaam Tanzania National Hospitals. | 298 HIV+ adults on ART aged 50+ | Cross-sectional study, Quantitative approach | Investigate polypharmacy, QoL and associated factors among older HIV-infected adults | Face-to-face interviewer-administered using the WHOQoL–HIV–BREF instrument. | Age, sex, polypharmacy, no of Co-morbidity, marital status, education | The general QoL |

FAHI - Function Assessment of HIV infection; HIV - Human Immunodeficiency Virus; HRQoL – Health-related quality of life; QoL – Quality of life; WHO – World Health Organisation

**Table S2: Correlates of Physical, Psychological, and Environmental of QoL**

| Article | Domains of QoL | | | | Correlates of QoL |
| --- | --- | --- | --- | --- | --- |
|  | Physical | Psychological | Social | Environmental |  |
| Karugaba, et al. ^23^ | VL suppression and illness status (self-reported) are strongly associated with QoL. The majority of participants (78.4%) who have reached VL reported good general QoL, with the highest scores in the Physical domain. | Good QoL is associated with a positive psychological state | The study revealed a low mean facet score of 56.92 for YALPH in satisfaction with sex life, highlighting the importance of romantic and sexual relationships in young adult development. | Overcrowded living arrangements are associated with poorer environmental QoL. | Socio-economic factors, higher education, and employment were associated with good HRQoL, Unsuppressed viral load and self-reported illness were associated with poor HRQoL. |
| Ndubuka, et al. ^24^ | Self-education about HIV-related issues was significantly associated with HRQoL physical scores. | High scores in this domain, positively correlated with QOL, self-education significantly enhanced psychological domain scores, | Poor scores were observed in the social relationships, possibly indicating experiences of stigma, discrimination, lack of family support, and insecure living conditions among PLWHA. | lowest scores were achieved in the environment domain (including physical safety, home environment, financial resources, health and social care accessibility, freedom, and participation in recreation. | Employment was associated with higher overall HRQoL scores, while educational levels did not show a significant relationship with HRQoL scores. |
| Okere, et al. ^25^ | Not studied. | The psychological or emotional domain of the QoL scored the lowest. Participants experienced low emotional/psychological QoL. | Like other QoL studies, the social domain in this study scored lowest owing to stigma and discrimination due to fear and lack of awareness as HIV continues to isolate those affected from meaningful relationships | Urban participants scored higher on QoL than rural participants due to better living conditions, increased HIV awareness, and anonymity, resulting in fewer stigmatising environments for HIV-positive people. | Service access factors contributed considerably to HRQoL among DSD participants.  Shorter time spent during clinic/club and the settings of service delivery were factors significantly associated with perceived HRQoL.  The study also revealed that QOL declines with age**.** As the PLHIV population on ART ages and comorbidities increase, emotional support becomes increasingly important.  Educational level, employment, and income level were not associated with QoL, |
| Perez, et al. ^26^ | QoL improvements persisted over 48 weeks postpartum in relation to Physical Health Summary (PHS) domains. | QoL improvements persisted over 48 weeks postpartum for Mental Health Summary (MHS) domains. | Not studied | Not studied. | Lower education levels negatively impact MHS scores, possibly due to poor socioeconomic outcomes. Internet use, television watching, and bank account ownership improve scores. Unemployment affects QoL among 62% of participants. |
| Surur, et al. ^27^ | The highest mean score was observed in the physical domain (mean ¼ 15.8, SD ¼ 3.5). The physical domain (measuring pain and discomfort, energy and fatigue, and sleep and rest) showed the highest mean QoL scores. Furthermore, self-education about HIV-related issues was significantly associated with QOL. | Most participants were satisfied with their physical health, had psychologically adjusted to their life on ART, and were able to manage their work and daily activities. | Not studied. | The lowest mean score was observed in the environment domain (mean ¼ 12.9, SD ¼ 2.5) due to the presence of stigma, discrimination and poor living conditions. | Employment was an essential factor associated  with overall QoL scores in participants. Educational levels were not associated with their HRQoL scores,  Self-education about HIV-related issues was significantly associated with QoL. |
| Wang, et al. ^28^ | Most participants reported no issues with mobility (95.7%), self-care (99.0%), and usual activities (96.9%). However, 32.9% experienced some problems with pain/discomfort.   - Lower quality of life linked to experiences of violence may be related to pain. | Lower QoL associated with the experience of violence may reflect the psychological effects of the violence.   - This study revealed that lower QoL among FSW living with HIV is associated with drug use, experience of violence, and moderate levels of internalised stigma. | The study found a high prevalence of homelessness and experience of stigma among participants (88%). Despite the lack of independent association between homelessness and QoL scores, homelessness was associated with increased odds of reporting mobility problems, pain/discomfort, and lower EQ-VAS scores. | Not studied | •Current ART use was independently associated with higher QoL   - Service access factors significantly impact HRQoL among DSD participants, with shorter clinic/club time and service delivery settings being significant. Age and comorbidities increase QoL - Educational, employment, and income levels do not correlate. |
| Mutabazi-Mwesigire, et al. ^17^ | An increase in Physical Health Summary (PHS) was observed from the baseline visit to the 6-month visit, and PHS also improved with an increase in the level of education.  The presence of HIV-related symptoms, particularly among those in WHO stage 3&4, was associated with poor QoL. | Mental health scores improved among PLWH on ART, with females showing lower scores. Low GPGI scores were associated with alcohol use and depression.  Alcohol use negatively affected QoL, while no significant association was found between hazardous drinking and QoL. | Not studied. | Not studied. | Higher global QoL and PHS were observed with increasing education levels among patients receiving ART. |
| Massawe, et al. ^18^ | Co-morbidities are not associated with poor QoL in this study, possibly due to differences in study settings and co-morbidities assessed compared to other studies. | Not studied. | Participants who reported poor QoL had lower social domain scores, which may have been impacted by HIV-related stigma and societal discrimination. in | Not studied. | No formal or primary education predicted poor QoL in the present study. |

AIDS - Acquired Immunodeficiency Syndrome; HIV - Human Immunodeficiency Virus; HRQoL – health-related quality of life; MHS – Mental Health Summary; PHS – Physical Health Summary; QoL – quality of life; WHO – World Health Organisation
